# A population-based analysis of clinical features and lymph node dissection in head and neck malignant neurogenic tumors

**DOI:** 10.1186/s12885-021-08307-4

**Published:** 2021-05-24

**Authors:** Xiaolian Fang, Shengcai Wang, Junyang Zhao, Yamei Zhang, Jie Zhang, Yanzhen Li, Xiaodan Li, Jun Tai, Xin Ni

**Affiliations:** 1 Department of Otolaryngology, Head and Neck Surgery, Beijing Children’s Hospital, Capital Medical University, National Center for Children’s Health, Beijing, 100045 China; 2 Department of Pediatric Oncology Center, Beijing Children’s Hospital, Capital Medical University, National Center for Children’s Health, Beijing, 100045 China; 3 Department of Otolaryngology, Children’s Hospital, Capital Institute of Pediatrics, Beijing, 100020 China

**Keywords:** Head and neck, Lymph node dissection, Overall survival, Neuroblastoma, Esthesioneuroblastoma

## Abstract

**Background:**

The influence of lymph node dissection (LND) on survival in patients with head and neck neurogenic tumors remains unclear. We aimed to determine the effect of LND on the outcomes of patients with head and neck neurogenic tumors.

**Methods:**

Data of patients with surgically treated head and neck neurogenic tumors were identified from the Surveillance, Epidemiology, and End Results (SEER) database (1975–2016) to investigate the relationship between LND and clinical outcomes by survival analysis. Subgroup analysis was performed in IVa and IVb group.

**Results:**

In total, 662 head and neck neurogenic tumor patients (median age: 49.0 [0–91.0] years) met the inclusion criteria, of whom 13.1% were in the IVa group and 86.9% were in the IVb group. The median follow-up time was 76.0 months (range: 6.0–336.0 months), and the 5-year and 10-year overall survival was 82.4% (95% CI, 0.79–0.85) and 69.0% (95% CI, 0.64–0.73). Cox regression analysis revealed older age (*P* < .001), advanced stage (*P* = .037), African American race (*P* = .002), diagnosis before 2004 (*P* < .001), and chemotherapy administration (*P* < .001) to be independent negative predictors of overall survival. Kaplan-Meier analysis demonstrated that LND was not a predictor of clinical nodal negativity (cN0) in either IVa or IVb patients.

**Conclusions:**

In head and neck neurogenic patients, LND may not impact the outcome of cN0 in either IVa or IVb group. These data can be recommended in guiding surgical plan and future studies.

## Background

Neurogenic tumors that originate from neural crest derivatives occur in the head and neck in 25–45% of cases [1–3]. Head and neck malignant neurogenic tumors commonly present as nasal obstructions, indolent masses and partly with Horner’s syndrome. They mainly include neuroblastoma, ganglioneuroblastoma (GNB), and esthesioneuroblastoma (ENB) [1, 4, 5]. According to the International Classification of Childhood Cancer (ICCC-3), patients are fell into group IV: neuroblastoma and other peripheral nervous cell tumors [6]. Neuroblastoma and GNB are both neuroblastic tumors and the former is more poorly differentiated [2]. ENB as a special neurogenic tumor entity appears to arise from the olfactory membrane of the sinonasal tract [1, 7].

As known for most head and neck cancers, the regional and distant metastases have been identified as one of the major challenges affecting long-term survival [8]. Thorough or selective neck dissection has been the criterion for patients with an advanced stage or positive lymph nodes in most cervical cancers [9, 10]. However, there is no consensus on the influence of lymph node dissection (LND) on the survival of patients with head and neck neurogenic tumors. A previous study showed that survival was not affected by the extent of resection of cervical neuroblastic tumors [11]. Moreover, Kuan et al. declared that the function of elective management of the cervical lymph nodes remains controversial in ENB [8]. Therefore, this study aims to investigate whether or not LND affects the outcomes of patients with head and neck malignant neurogenic tumors by using population data obtained from the Surveillance, Epidemiology, and End Results (SEER) database. Furthermore, we aimed to identify predictive factors for long-term outcomes.

## Methods

### Data source

This retrospective study used data from the National Cancer Institute’s SEER project database from 1975 to 2016. The study protocol was designed according to the Strengthening the Reporting of Observational Studies in Epidemiology (STROBE) statement [12]. Institutional review board approval was waived for SEER studies, as the database was identified and available for public use.

The SEER 18 database was searched to identify all patients diagnosed with neuroblastoma and peripheral nervous cell tumors using the 3rd edition of the ICCC site recode extended classification and the IARC code IV. Site codes for tumors originating from the peripheral nervous system and soft tissue of the head, face, neck, nasal cavity, or paranasal sinuses were searched. The following categories were obtained from the data: age at diagnosis, sex, ethnicity, year of diagnosis, SEER historic stage, reason for not undergoing cancer-directed surgery, survival months, vital status recode, SEER classification for other causes of death, therapy, examination of regional nodes and their respective positivity status. Detailed information on all variables mentioned in the SEER database is available in Appendix C of the SEER manual.

### Patient selection

The inclusion criteria were primary diagnosis of malignant neurogenic tumor and definitive surgery performed at the primary site. Only patients followed for 6 months were included in this study. The most standard classification of neuroblastoma, called the International Neuroblastoma Staging System (INSS), was not included in the SEER database, and the SEER historic stage was used to obtain the tumor stage. Therefore, disease classification included localized, regional, and distant subgroups. Data on age, sex, ethnicity, tumor size, survival months, and therapy were obtained directly from the original data without further conversion. Overall survival (OS) data for patients in the study cohort were based on vital status, in which patients were coded as alive or dead. Disease-specific survival (DSS) data were obtained according to other causes of death. The clinical nodal stage of clinical nodal negative (cN0) patients and clinical nodal positive (cN+) patients was based on criteria taken from the “SEER historic stage A”, “SEER Combined Summary Stage 2000”, and “Derived AJCC Stage Group, 7th edition”. The diagnosis was determined on imaging and physical examination. According to the number of examined lymph nodes, we divided the patients into two groups: the non-LND and the LND group. The pathology finding of nodes was obtained on “Regional nodes positive”. Regarding the years of diagnosis, 3 time periods were considered as follows: before 1998, 1998–2004 and after 2004. This was done because the study period was too long to ensure that the treatment protocols would remain consistent throughout its entirety.

### Statistics

Statistical analysis was performed using SPSS version 25.0 and Stata software version 12.0. A normally distributed test was performed on quantitative data. Normally distributed measurement data are expressed as X ± S, while the non-normally distributed measurement data are expressed as medians (interquartile range). Categorical variables were compared using the χ^2^ test. The correlation between the classification variables was determined using Spearman’s correlation test or the Mann-Whitney U test. The kappa index (kappa) was used to assess the agreement between two clustering results. Kaplan-Meier survival analysis was performed to estimate OS and DSS. The log-rank test or Breslow test were used to determine statistically significant predictors of survival on univariate analysis. Covariates with *P* < 0.05 in the univariate analysis or important covariates were included in the multivariate model. Multivariate Cox analysis was performed to estimate the independent effects of age, treatment and lymphadenectomy to obtain hazard ratios (HR) and 95% confidence intervals (CI). A *P*-value of < 0.05 was considered statistically significant.

## Results

### Population features

**Fig. 1:**
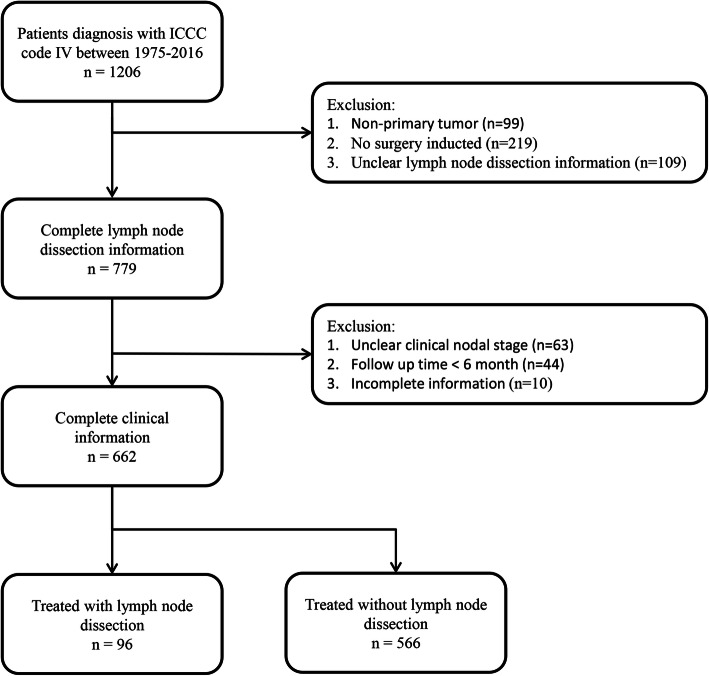
Flowchart for patient information retrieval from the Surveillance, Epidemiology, and End Results database

**Fig. 2:**
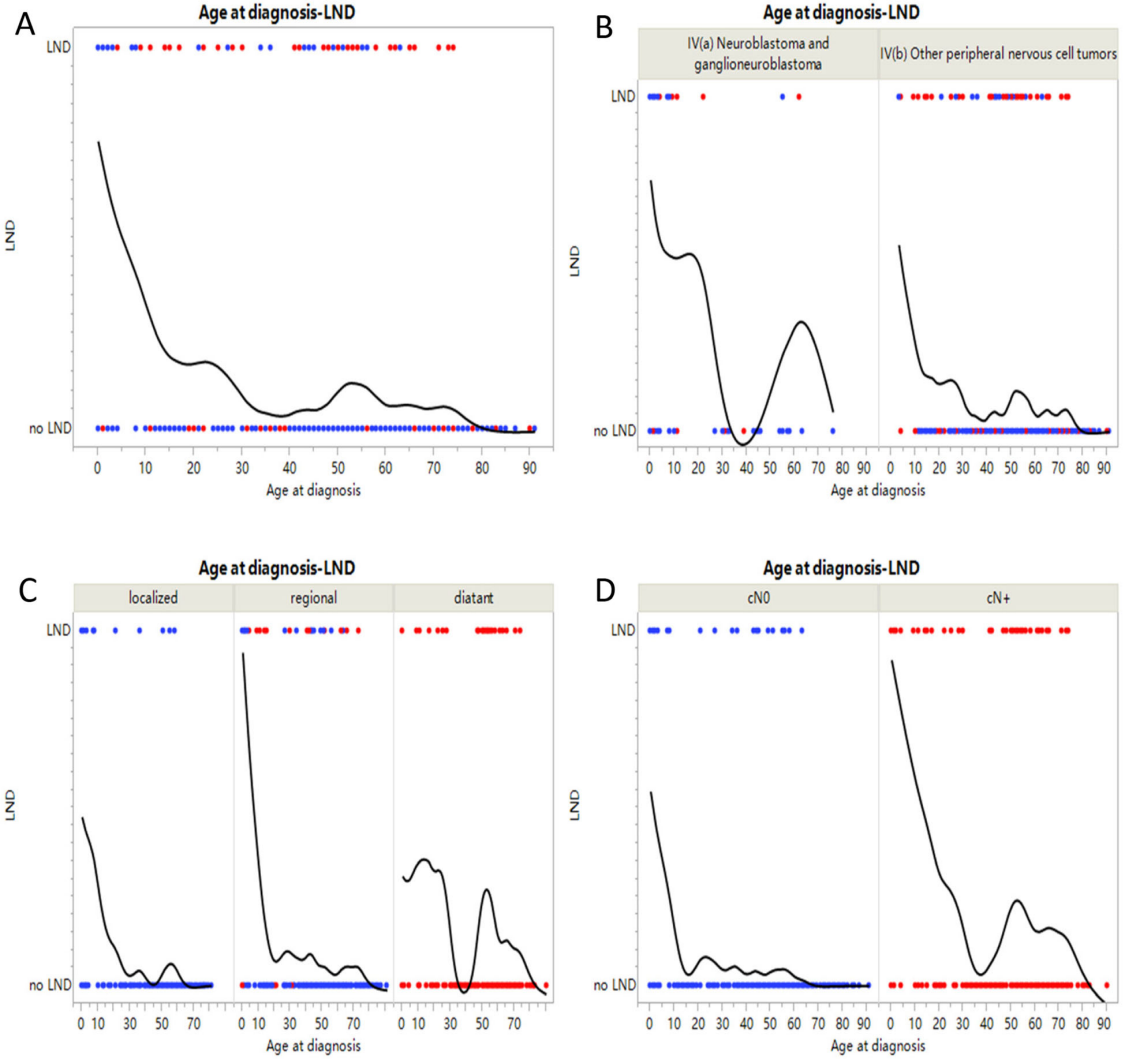
The distribution between age at diagnosis, histology and LND

A total of 662 patients diagnosed with head and neck neurogenic tumors between 1975 and 2016 were included. A total of 544 cases were excluded in which 99 cases were patients with non-primary tumor, 219 were patients who did not received surgery, 109 were patients with unclear LND information, 63 were patients with unclear clinical nodal stage, and 54 were patients with incomplete information (Fig. 1). The patient demographic characteristics are shown in Table 1. Among them, 293 (44.3%) patients were female and 369 (55.7%) were male. In total, 81.3% of patients were white, 8.0% were African American, and 10.7% were another race or ethnicity. In addition, 572 (86.4%) patients were Non-Spanish-Hispanic-Latino, and 90 (13.6%) patients were Spanish-Hispanic-Latino. The median age at diagnosis was 49.0 years old (range: 0–91.0 years) and 99 (15.0%) patients were children. According to the SEER stage, 198 (29.9%) patients had localized tumors, 294 (44.4%) had regional tumors, and 170 (25.7%) had distant tumors. The common primary sites were nasal cavity (573/662, 86.7%), and soft tissue (81/662, 12.2%). According to the classification of ICCC stage, 87 (13.1%) patients were in stage IVa and 575 (86.9%) patients were in stage IVb. The distribution between age at diagnosis, histology and LND is shown in Fig. 2.

**Table 1:** Demographic characteristics and clinical features of the population in our cohort

Variables	Total (***n*** = 662)	%	IVa (***n*** = 87)	%	IVb (***n*** = 575)	%
**Age at diagnosis (years)**	49.0 (0–91.0)		1.0 (0–76.0)		51.0 (4–91.0)	
Children	99	15.0	70	80.5	29	5.0
Adults	563	85.0	17	19.5	546	95.0
**Sex (Female/Male)**						
Female	293	44.3	45	51.7	248	43.1
male	369	55.7	42	48.3	327	56.9
**Race (White, Black, Other)**						
White	538	81.3	71	81.6	467	81.2
Black	53	8.0	9	10.3	44	7.7
others	71	10.7	7	8.0	64	11.1
**Origin**						
Non-Spanish-Hispanic-Latino	572	86.4	73	83.9	499	86.8
Spanish-Hispanic-Latino	90	13.6	14	16.1	76	13.2
**ICD-O-3 Histology/behavior, malignant**						
**IV(a) Neuroblastoma and ganglioneuroblastoma**	87	13.1				
9500/3:Neuroblastoma	74	11.2	74	85.1		
9490/3:Ganglioneuroblastoma (GNB)	13	1.9	13	14.9		
**IV(b) Other peripheral nervous cell tumors**	575	86.9				
8680/3: Paraganglioma, malignant	14	2.1			14	2.4
9522/3:Esthesioneuroblastoma (ENB)	559	84.4			559	97.2
9501/3:Medulloepithelioma,malignant	1	0.2			1	0.2
9503/3: Neuroepithelioma, malignant	1	0.2			1	0.2
**Primary Site labeled**						
C30.0-Nasal cavity	451	68.1	6	6.9	445	77.5
C31.0–31.9-Paranasal sinus	119	18.0	8	9.2	111	19.3
C47.0-Periph nerves & autonomic nervous sys: head, face, neck	45	6.8	39	44.8	6	1.0
C49.0-Conn, subcutaneous, other soft tis: head, face, neck	36	5.4	30	34.5	6	1.0
others	11	1.7	4	4.6	7	1.2
**Diagnosis years**						
< 1998	71	10.7	16	18.4	55	9.6
1998–2004	147	22.2	20	23.0	127	22.1
> 2004	444	67.1	51	58.6	393	68.3
**SEER historic stage**						
Localized	198	29.9	31	35.6	167	29.0
regional	294	44.4	47	54.0	247	43.0
distant	170	25.7	9	10.3	161	28.0
**Clinical Nodal status (cN0/cN+)**						
cN0	420	63.4	43	49.4	377	65.6
cN+	240	36.6	44	50.6	198	34.4
**Radiation therapy (No/Yes)**						
No	239	36.1	71	81.6	168	29.2
Yes	423	63.9	16	18.4	407	70.8
**Chemotherapy (No/Yes)**						
No	482	72.8	58	66.7	424	73.7
Yes	180	27.2	29	33.3	151	26.3
**Lymph node dissection (No/Yes)**						
No	566	85.5	38	43.7	528	91.8
Yes	96	14.5	49	56.3	47	8.2
**Follow-up time (months)**	76.0 (6.0–336.0)		95.0 (6.0–317.0)		74.0 (6.0–336.0)

In addition, 63.4% (420/662) patients were cN0 and 36.6% (242/662) were cN+. The rate of LND was 14.5% (96/662), the LND rate for IVa group was 56.3% (49/87) and 8.2% (47/575) in IVb group (*P* < .001). Overall, 72.9% (70/96) patients with LND had pathologically positive nodes. After Kappa analyses showed that preoperative lymph node staging and postoperative lymph node staging observer agreements were excellent (Kapper = 0.829, *P* < .001).

### Survival analysis

**Fig. 3:**
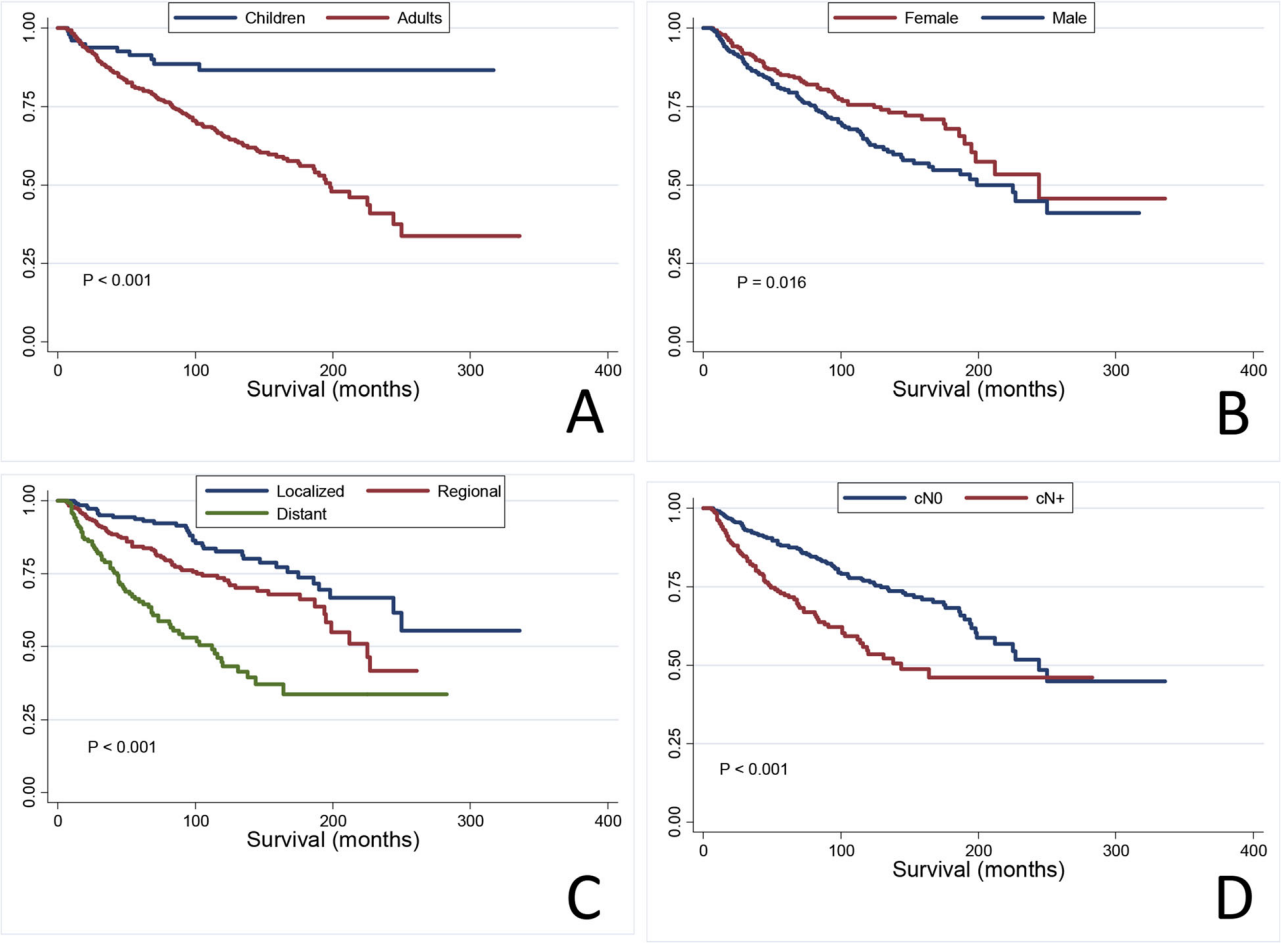
Survival curves of OS and DSS of whole patients

Overall, the median follow-up time was 76.0 months (range: 6.0–336.0 months), and 633 (95.6%) patients were followed up over 1 year and 387 (58.5%) patients were followed up over 5 years. The 5-year and 10-year OSs were 82.4% (95% CI, 0.79–0.85) and 69.0% (95% CI, 0.64–0.73), respectively. The 5-year and 10-year DSSs were 87.5% (95% CI, 0.84–0.89) and 79.1% (95% CI, 0.75–0.83), respectively. The OS and DSS were significant better in children than in adults (88.9% vs. 69.6%, *P* < .001, *P* = .011). Similarly, women had a higher OS and DSS rates than men (76.8% vs. 69.1%, *P* = 0.016, *P* = 0.026). In addition, SEER historic stage and clinical nodal status were both significant predictors of outcomes in our cohort (*P* < 0.001, *P* < 0.001). Patients diagnosed between 1998 and 2004 and after 2004 had better outcomes than those diagnosed before 1998 (82.7% vs. 59.9% vs. 35.2%, *P* = .008, *P* = .025). The OS and DSS rates did not differ between patients who received LND and patients who did not (71.7% vs 77.1%, *P* = 0.325, *P* = 0.997). In the IVa disease group, the OS and DSS rates were better in patients with LND than patients with non-LND (98.0% vs 84.2%, *P* = .01, *P* = .018). However, the IVb group showed opposite results: OS and DSS rates were better in the patients without LND (66.0% vs 82.2%, *P* = .002, *P* < .001) (Fig. 3). Moreover, the overall survival and DSS in patients who received chemotherapy or radiation-therapy was worse than not (*P* < .001, *P* < .001). Comparative analysis found that patients who received chemotherapy or radiation had more advanced stages of the disease (*P* < .001, *P* < .001). Multivariable Cox regression analysis (Table 2) revealed older age (*P* < .001), advanced stage (*P* = .037), African American race (*P* = .002), diagnosis before 2004 (*P* < .001), and chemotherapy administration (*P* < .001) to be independent negative predictors of OS. The risk of outcomes increased 1.04-fold per year with increasing age (95% CI = 1.030–1.053).

**Table 2:** Cox regression analysis of overall survival and disease-specific survival of the entire cohort

	Overall Survival		Disease-Specific Survival	
Variables	OR(95%CI)	***P*** Value	OR(95%CI)	***P*** Value
**Age at diagnosis (years)**	1.041 (1.030–1.053)	**< 0.001**	1.017 (1.006–1.028)	**0.002**
**Sex (Female/Male)**	1.309 (0.961–1.783)	0.087	1.307 (0.886–1.928)	0.177
**Race**				
Others	1(Reference)		1(Reference)	
White	1.441 (0.844–2.458)	0.181	1.265 (0.654–2.445)	0.485
Black	2.879 (1.494–5.550)	**0.002**	2.392 (1.068–5.355)	**0.034**
**Diagnosis**				
< 1998	1(Reference)		1(Reference)	
1998–2004	1.560 (1.080–2.255)	**0.018**	1.855 (1.194–2.882)	**0.006**
≥ 2004	2.630 (1.743–3.967)	**< 0.001**	2.742 (1.632–4.607)	**< 0.001**
**SEER stage**				
Localized	1(Reference)		1(Reference)	
Regional	1.613 (1.056–2.465)	**0.027**	2.696 (1.427–5.095)	**0.002**
Advanced	2.051 (0.909–4.626)	0.084	4.043 (1.374–11.900)	**0.011**
**Clinical node status (cN0/cN+)**	1.637 (0.801–3.345)	0.177	1.480 (0.606–3.616)	0.390
**Histology (IVa/IVb)**	1.697 (0.827–3.482)	0.149	2.836 (1.108–7.260)	**0.030**
**Chemotherapy (No/Yes)**	2.041 (1.465–2.845)	**< 0.001**	2.147 (1.433–3.219)	**< 0.001**
**Radiation (No/Yes)**	0.869 (0.643–1.175)	0.362	1.134 (0.760–1.692)	0.538
**Lymph node detection (No/Yes)**	1.250 (0.756–2.066)	0.384	1.231 (0.679–2.231)	0.494

*CI* Confidence interval

### Subgroup survival analysis by histology

**Fig. 4:**
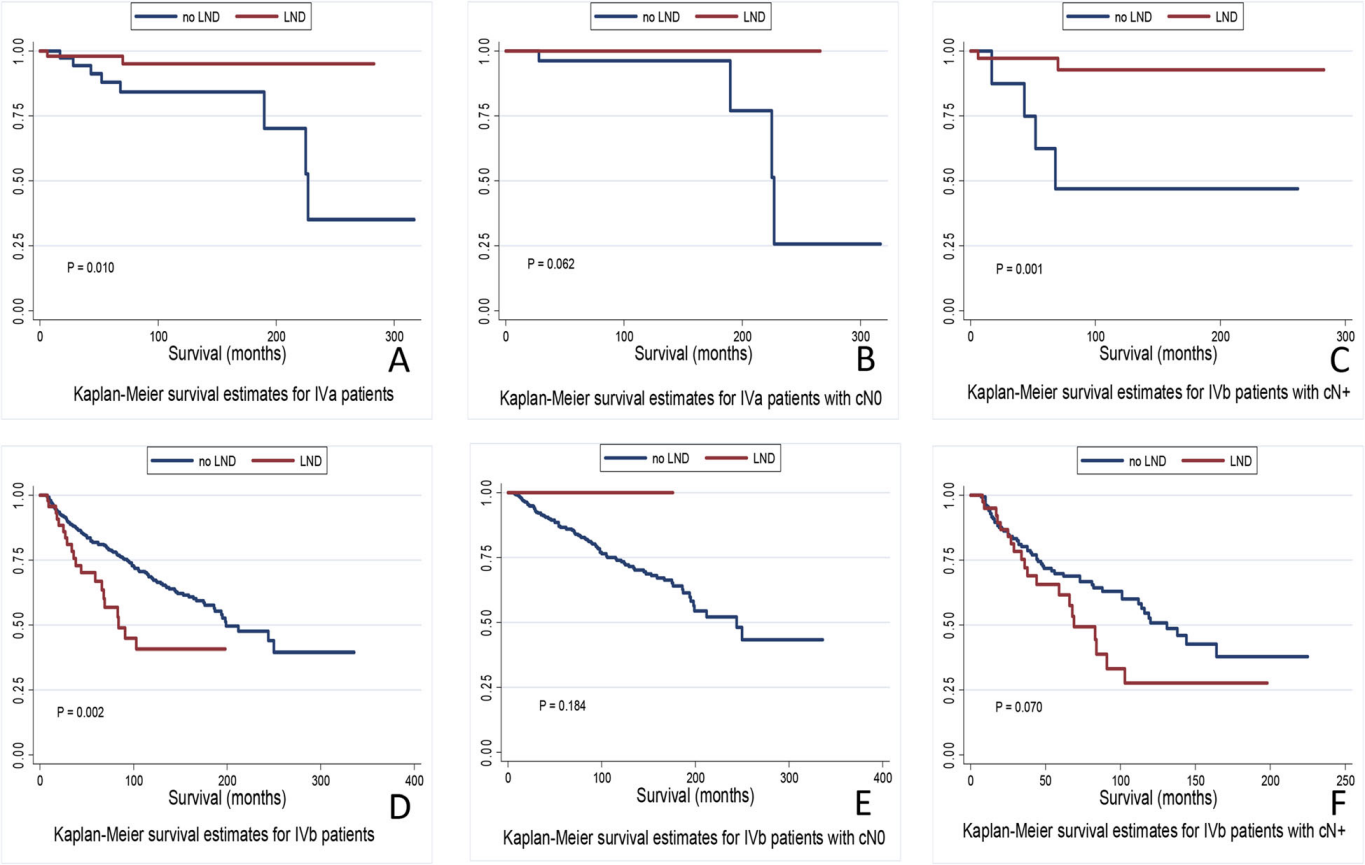
Survival curves of OS and DSS based on lymph node dissection

For stage IVa patients, Kaplan-Meier analysis demonstrated that LND was a positive predictor of OS and DSS in the whole cohort or in cN+ patients (*P* = .001, *P* < .001, respectively) but not in cN0 patients (*P* = .062, *P* = .194). Univariable analysis revealed that children (*P* = .007), SEER stage (*P* < .001), diagnosis after 1998 (*P* < 0.023), LND (*P* = .018), and radiation administration (*P* < .001) were predictors of OS and DSS. Multivariable analysis revealed that cN+ (*P* = .005), and LND (*P* = .024) were independent predictors of OS and DSS (Table 3) **(**Fig. 4**)**.

**Table 3:** Univariate and multivariate analysis of overall survival in type IVa and IVb patients

	IVa patients				IVb patients			
	Univariate analysis		Multivariate analysis		Univariate analyses		Multivariate analysis	
Variables	KM analysis	***P*** Value	OR(95% CI)	***P*** Value	KM analysis	***P*** Value	OR(95% CI)	***P*** Value
Age at diagnosis (Child/Adult)	10.494	**0.001**	1.440 (0.203–10.229)	0.715	0.851	0.356		
Sex (Female/Male)	0.075	0.785			5.691	**0.017**	1.279 (0.929–1.762)	0.132
Diagnosis year	4.734	0.094			11.611	**0.003**	1	
< 1998/1998–2004							1.350 (0.932–1.956)	0.112
< 1998/≥2004							2.544 (1.669–3.876)	**< 0.001**
Race	1.228	0.541			10.651	**0.005**	1	
Others/White							1.214 (0.710–2.076)	0.479
Others/Black							2.365 (1.214–4.606)	**0.011**
Clinical node stage (cN0/cN+)	0.518	0.471	296.560 (3.493–25,180.669)	**0.012**	34.543	**< 0.001**	1.403 (0.654–3.011)	**0.384**
SEER stage	9.727	**0.008**	1		43.991	**< 0.001**	1	
Localized/Regional			0.483 (0.044–5.314)	**0.552**			1.674 (1.090–2.572)	**0.019**
Localized/Distant			0.016 (0.000–1.982)	**0.093**			2.282 (0.961–5.421)	**0.062**
Radiation therapy (No/Yes)	24.005	**< 0.001**	79.660 (1.931–3285.577)	**0.021**	1.012	0.292		
Chemotherapy (No/Yes)	0.134	0.714			27.402	**< 0.001**	1.669 (1.187–2.348)	**0.003**
Lymph node dissection (No/Yes)	6.642	**0.010**	1.090 (0.422–2.818)	**0.013**	9.604	**0.002**	1.420 (0.849–2.376)	0.182

For patients with IVb, Kaplan-Meier analysis demonstrated LND was negative predictor of overall survival and DSS in whole patients but not patients with cN+ (*P* = .070, *P* = .069) or cN0 patients (*P* = .184, *P* = .330). Multivariable analysis revealed SEER stage (*P* = .017), diagnosis year (*P* = .001), and received chemotherapy (*P* < .001) to be independent predictors of OS and DSS.

## Discussion

Neurogenic tumors are genetically heterogeneous in prognosis and easily metastasize to the lymph nodes, bone marrow, bones, liver, and skin [13–15]. Neurogenic tumors with head and neck origin remain extremely rare, with relatively better outcomes [16]. Although total surgical resection with neck dissection for most head and neck cancers is the standard surgical therapy, LND is controversial in head and neck neurogenic tumors [17–19]. A particular controversy is the prognostic significance of neck dissection in cN0 patients. To our knowledge, this is the first population-based study to analyze the association between neck dissection and survival in head and neck neurogenic tumors and represents the largest cohort to date.

Our demographic data demonstrated that the overall rate of LND was 14.5%, while the rates for the stage IVa and IVb groups individually were 56.3% (49/87) and 8.2% (47/575), respectively. The overall LND rate in the cohort was relatively low, regardless of whether the clinical stage was positive or negative. Our study also showed that LND did not improve OS or DSS in cN0 patients in stages IVa or IVb. This finding was consistent with other head and neck cancers reported in the literature, including cervical adenoid cystic carcinoma, ENB, and cervical unknown primary site of squamous cell carcinoma [19–21]. Additionally, a more minimally aggressive surgical approach was suggested for patients with cervical neuroblastic tumors [11]. Notably, cervical primary site diseases are clinically and biologically more favorable than abdominal neurogenic tumors [14, 16]. Therefore, a more intense lymphadenectomy may cause worse outcomes and complications in head and neck neurogenic tumors with cN0. Importantly, neck dissection was necessary for patients who were cN+ in IVa patients based on our primary findings.

However, it is odd that most (68.6%) cN+ patients did not get neck dissection in our cohort. And 79.8% (158/198) IVb patients with cN+ but only 18.1% (8/44) IVa patients with cN+ did not received LND by further analysis. This is related to the heterogeneity of two diseases, including the preferred treatment method and sensitivity to chemotherapy or radiotherapy. Except for individuals who refused to receive complete or elective neck dissection, some ENB patients may have received radiation therapy of the neck [5]. The selection of surgical approaches, including racial neck dissection and selective neck dissection, is worth further discussing in regards to cN+ patients.

The clinical features and outcomes varied greatly according to the histology of neurogenic tumors [14]. Our series found that IVa and IVb patients were significantly different in age between diagnosis and survival. The ENB was usually located in the nasal cavity, paranasal sinus and nasopharynx, and most frequently occurred in the fifth to sixth decades of life [5, 7, 22]. The cervical GNB or neuroblastoma originated from peripheral nerves or soft tissues and 90% were occurred in children younger than 10 years of age [16, 23]. In our study, the OS and DSS of children were better those adults. The age at diagnosis was an independent factor associated with outcomes and the risk of OS increased by 4.4% per year. Significant differences in prognosis existed between children and adults (*P* < .001). The age at diagnosis of neuroblastoma is a standard indicator of prognosis [23]. While young infants with favorable biological characteristics may have spontaneous regression, children diagnosed at over 1 year of age have a higher frequency of metastatic disease, need stricter treatment, and have a worse prognosis [23]. A retrospectively analyze of ENB cohort, the risk of overall death and cancer-specific death increased 3.1 and 1.6% per year [22]. In addition, malignant neurogenic tumors occurred slightly more commonly in males, and the male-to-female ratio was 1.3 in our series [24–26]. Moreover, the prognosis of male patients was worse than female patients by univariable analysis in our cohort. However, this was not true when considering the results of multivariable or subgroup analysis. This viewpoint is still controversial and needs more verification, especially in ENB patients [25].

Advanced-stage neurogenic tumors have a high metastasis rate and poor prognoses [27]. The SEER stage in the database categorized the patients into localized, regional, and distant groups. Though the INSS and Kadish stage, most used stage system for neuroblastoma and ENB, were not recorded in the database, our study showed that advanced stage classified by SEER stage was a significantly negative independent factor for both OS and DSS. Thus, the SEER stage had a good predictive value for outcomes in head and neck neurogenic tumors. Moreover, according to the Kappa identity test, the clinical node stage was also a good predictor of lymph node status. Further mining and analysis of data from the SEER database will provide more reliable results.

Regarding multimodal treatments, patients who received chemotherapy or radiation had a worse prognosis. This might be related to the advanced disease stage of patients in the chemo/radiation group. In addition, patient prognosis improved from 1975 to 2017 due to the significant evolution in the management of oncologic treatment and dramatic escalations in the intensity of therapy during these years [17]. Importantly, 1998 and 2004 were high frequency time nodes in the SEER database. Our data showed that patients diagnosed after 1998 and after 2004 had a better prognosis. This may be related to early diagnosis which ensured greater awareness and more standardized treatments of the disease in recent years. The substantial improvement in the prognosis of neuroblastoma patients during the past few decades may be attributed to improved recovery among patients with more benign forms of the disease who benefited from early diagnosis [17, 28].

The major limitation of this study is that no further analysis was performed in the subgroup limited to the small number of cases, especially the IVa group. The two most common diseases in the cohort were neuroblastoma and ENB and they are very different in terms of clinical features and outcomes. In addition, the reliability of our results could be improved by verifying our own data further. Moreover, we were also unable to report on other important variables like the extent of neck dissection and surgical approach, which were not recorded in the SEER database.

## Conclusions

Our study demonstrated that older age, advanced SEER stage, Asian American race, diagnosis before 2004, and chemotherapy administration significantly influenced the outcomes of head and neck malignant neurogenic tumors. Moreover, LND was not a predictor of OS and DSS in cN0 patients. These data can be recommended in guiding surgical plan and future studies. Larger prospective cohort studies are required to confirm our findings and determine the true prognostic impact of neck dissection. Further studies on different lymphadenectomy methods for cN+ patients are also needed.

## Data Availability

The data that support the conclusions of this article are available from the Surveillance, Epidemiology, and End Results database: Surveillance, Epidemiology, and End Results Program (https://seer.cancer.gov) SEER*Stat Database.

## Abbreviations

LND: Lymph node dissection
SEER: Surveillance, Epidemiology, and End Results
GNB: Ganglioneuroblastomam
ENB: Esthesioneuroblastoma
ICCC-3: International Classification of Childhood Cancer
STROBE: Strengthening the Reporting of Observational Studies in Epidemiology
INSS: International Neuroblastoma Staging System
OS: Overall survival
DSS: Disease-specific survival
CI: Confidence interval
HR: Hazard ratio
